# A retrospective analysis of the clinical efficacy of pancreatic duct stent implantation in the management of acute biliary pancreatitis requiring ERCP

**DOI:** 10.1186/s40001-023-01557-x

**Published:** 2023-12-15

**Authors:** Bo Peng, Zuoquan Wang, Chengsi Zhao, Genwang Wang, Di Liu, Tongtong Dong, Jinping Shi, Zuozheng Wang, Weijie Yao

**Affiliations:** 1 General Surgery Center, People’s Hospital of Ningxia Hui Autonomous Region, Yinchuan, Ningxia China; 2 General Surgery, The Third Affiliated Hospital of Xi’an Medical University, Xi ‘an, Shaanxi China; 3 Hepatobiliary Surgery, General Hospital of Ningxia Medical University, No. 804, Shengli South Street, Xingqing District, Yinchuan, 750004 Ningxia China; 4 School of Clinical Medicine, Ningxia Medical University, Yinchuan, Ningxia China

**Keywords:** Biliary pancreatitis, Endoscopy, Pancreatic duct stenting, Endoscopic retrograde cholangiopancreatography, Endoscopic nasobiliary drainage

## Abstract

**Background:**

This study aimed to investigate the feasibility, effectiveness, and safety of pancreatic duct stenting in managing acute biliary pancreatitis (ABP) necessitating endoscopic retrograde cholangiopancreatography (ERCP). It further aimed to provide valuable insights for subsequent clinical diagnosis and treatment.

**Methods:**

This research employs an observational retrospective case–control study design, encompassing patients with ABP who underwent ERCP at the hepatobiliary surgery department of the General Hospital of Ningxia Medical University between August 1, 2018, and December 31, 2020. A total of 229 cases were screened based on inclusion and exclusion criteria. Regardless of ABP severity, patients were categorized into the stent group (141) and the non-stent group (88). Changes in blood amylase (Amy), lipase (LIP), leukocyte count (WBC), total bilirubin (TBIL), alanine aminotransferase (ALT), hematocrit (HCT), and creatinine (CR) were compared between the two groups. Moreover, variables such as recovery time for oral feeding, hospitalization duration, hospitalization costs, local complications, systemic complications, and new organ failure were recorded to assess the therapeutic effect of pancreatic duct stenting.

**Results:**

No significant differences were observed in gender, age, Acute Physiology and Chronic Health Evaluation (APACHE) II score, ABP severity grade, organ failure (OF), cholangitis, or biliary obstruction between the pancreatic stent and non-stent groups (*P* > 0.05). There was no significant difference in the incidence of complications related to acute pancreatitis between the two groups (*P* > 0.05). The median fasting and hospitalization times of patients in the stent group were significantly shorter than those in the non-stent group (*P* < 0.05). No significant differences between the groups were observed in hospitalization costs and in-hospital mortality (*P* > 0.05). There were no significant variations in white blood cell (WBC) count, TBIL, ALT, and creatinine (Cr) at admission, 72 h, and in the differences between the two groups (*P* > 0.05). The levels of Amy at admission and 72 h in the stent group were significantly higher than those in the non-stent group (*P* < 0.05). The differences in LIP and HCT in the stent group were considerably higher than in the non-stent group (*P* < 0.05). Although no significant differences were observed in mean Amy and LIP between the two groups (*P* > 0.05), the mean 72-h HCT in the stent group was 38.39% (95% confidence interval [CI] 37.82%–38.96%) was lower than that in the non-stent group (39.44%, 95% CI 38.70–40.17%) (*P* < 0.05).

**Conclusion:**

In the stent group, feeding time and hospital stay were significantly shorter than those in the non-stent group. No significant differences were observed between the two groups in the incidence of complications and mortality. The HCT value decreased more rapidly in the stent group. Early pancreatic stent implantation demonstrated the potential to shorten the eating and hospitalization duration of patients with ABP, facilitating their prompt recovery.

*Trial Registration*: This study was registered as a single-center, retrospective case series (ChiCTR1800019734) at chictr.org.cn.

## Introduction

Acute pancreatitis (AP) stands out as a prevalent digestive system disorder. In recent years, its incidence has seen a steady rise, attributed to advancements in living standards and shifts in dietary patterns. Beyond posing a threat to human health, AP imposes substantial economic burdens. Reports indicate that AP-related medical expenses in European and American nations surpass 2.6 billion US dollars annually [1]. While the majority of AP incidents manifest as mild cases with no organ failure (OF) or complications, resolving within a week of medical intervention, some patients experience severe acute pancreatitis (SAP). SAP is characterized by prolonged (> 48 h) OF, posing a significant threat to multiple systems, such as respiration, circulation, and digestion, with mortality rates ranging from 15 to 30% [2]. The causative factors behind SAP-led OF remain inadequately elucidated. Early OF is frequently linked to aseptic inflammation, resulting in high mortality. In instances where septicemia follows infectious pancreatic necrosis, late-stage OF may also ensue [3]. Consequently, SAP necessitates comprehensive interdisciplinary management.

The etiology of AP is diverse, exhibiting regional variations. In developed nations, alcoholism and cholelithiasis account for the majority of cases (36%). Conversely, cholelithiasis is the primary culprit in China, contributing to approximately 40–70% of AP incidence rates [4]. Despite extensive investigation, the pathogenesis of acute biliary pancreatitis (ABP) remains incompletely understood. The interplay of anatomy, genetics, biliary factors, and pancreatic duct obstruction is presumed to contribute to the onset of this condition. As far back as a century ago, Opie et al. proposed the “common pathway theory”, positing that bile reflux resulting from bile duct obstruction served as the initiating event for ABP. Fifty years later, Acosta and Ledesma introduced the “gallstone passage theory”, suggesting that the onset of ABP was triggered by the abnormal stimulation of transitional stones traversing the common channel of the biliary and pancreatic duct [5]. Both theories emphasize that following bile duct obstruction, the pressure in the bile duct exceeds normal levels, leading to the retrograde flow of bile or duodenal fluid into the pancreatic duct. The retrograde flow overly stimulates and activates pancreatic enzymes, resulting in excessive self-digestion of the pancreas and the occurrence of ABP [6]. Nipple edema or Oddi sphincter spasm induced by temporary stone obstruction is crucial in developing ABP [7]. Despite ongoing debates surrounding the pathogenesis of ABP, the prevailing consensus supports the “common channel” theory. This theory asserts that the merging and opening of the bile duct and pancreatic duct to the duodenal papilla form a common channel. Any factor causing common bile duct obstruction, mainly stones, can elevate the pressure of the bile and pancreatic ducts. The reflux of bile and its cytokines then stimulates the pancreas, triggering the abnormal activation of trypsin and leading to AP [8].

Conservative medical treatment and surgical intervention represent common strategies for managing ABP. Laparoscopic cholecystectomy serves as a frequently employed surgical approach; however, patients commonly experience abdominal and shoulder pain post-laparoscopy, primarily attributed to residual blood in the abdominal cavity and carbon dioxide (CO_2)_ pneumoperitoneum. As an endoscopic procedure involving the implantation of a plastic pancreatic duct stent, pancreatic duct stenting offers a solution to enhance pancreatic drainage, reduce CO_2_ residue, and effectively address biliopancreatic duct obstruction [9]. This intervention alleviates the clinical symptoms of pancreatitis in patients, reducing the likelihood of recurrence. There are limited clinical studies on using pancreatic duct stenting to treat patients with ABP. Therefore, this study aims to observe the clinical effectiveness of pancreatic duct stenting in ABP treatment, providing a valuable reference for its management.

## Methods

### General information

This observational retrospective case–control study enrolled patients with ABP who underwent endoscopic retrograde cholangiopancreatography (ERCP) in the hepatobiliary surgery department of the General Hospital of Ningxia Medical University from August 1, 2018, to December 31, 2020. A total of 229 cases were screened based on inclusion and exclusion criteria. Irrespective of ABP severity, patients were categorized into the stent group (141) and the non-stent group (88). This study adhered to ethical standards outlined in the Declaration of Helsinki (2013 revision), approved by the ethics committee of the General Hospital of Ningxia Medical University (No.: [2018]0725A). All patients provided informed consent before ERCP, granting permission to use clinical data in subsequent research.

### Grouping

Patients in the pancreatic duct stent group underwent ERCP within 72 h after admission, with pancreatic duct stents placed during the operation. The decision to perform biliary lithotomy or endoscopic sphincterotomy (EST) was determined based on intraoperative cholangiography. Patients in the non-stent group received conservative treatment post-admission. The timing of ERCP was based on their condition, without placing pancreatic stents during the operation. The timing and indications of ERCP in the non-pancreatic stent group aligned with the following American College of Gastroenterology (ACG) guidelines for AP treatment: [10] I. Patients with AP complicated by acute cholangitis should undergo ERCP within 24 h of admission. II. Emergency ERCP is not required without laboratory or clinical evidence of persistent biliary obstruction. III. If common bile duct stones are highly suspected in the absence of cholangitis, jaundice, or both, ERCP should be performed after confirmation by magnetic resonance cholangiography (MRCP) or endoscopic ultrasonography (EUS).

### Inclusion criteria

I. Age ≥ 18 years old. II. A diagnosis consistent with ABP [11]. III. Onset time ≤ 72 h. IV. Complete clinical case data (diagnosis, auxiliary examination, and operation records).

### Exclusion criteria

I. AP during pregnancy. II. Contraindications related to endoscopic surgery. II. Acute attack of chronic pancreatitis. IV. Previous ERCP due to AP. V. AP caused by drinking habits and hyperlipidemia or other metabolic diseases. VI. Incomplete clinical data.

### Diagnostic criteria for AP [12]

I Presence of typical epigastric pain in AP. II Blood amylase, lipase concentrations, or both exceeding three times the upper limit of normal. III Enhanced CT or MRI features indicative of AP. Clinically, AP can be diagnosed if at least two of the above three criteria are met.

### Diagnostic criteria for ABP [11]

I. Manifestation of typical upper abdominal pain symptoms associated with AP. II. Imaging examinations (such as abdominal CT and B-mode ultrasound) revealing the presence of stones in the biliary system or acute cholecystitis. III. Elevated blood amylase, urine amylase levels, or both exceeding three times the normal value. IV. Abnormal total serum bilirubin (TBIL) levels, liver function, or both. V. Absence of other diseases causing elevated levels of TBIL, amylase, or aspartate aminotransferase (AST)/alanine transaminase (ALT).

### Diagnostic criteria for complications

#### Systemic complications and organ dysfunction

I. Systemic inflammatory response syndrome (SIRS): body temperature > 38 ℃ or < 36 ℃, heart rate > 90 beats/min, breathing > 20 times/min, or PaCO_2_ < 4.3kpa (32 mmHg). Leukocyte count > 12 × 10^9^/L or < 4 × 10^9^/L or immature neutrophils > 10%. Diagnosis requires meeting the above two requirements. II. Sepsis: presence of pathogen evidence and meeting the diagnosis of SIRS. III. Acute OF: occurs if any one organ fails or has a modified Marshall score > 2. IV. Multiple organ dysfunction syndrome (MODS): according to the modified Marshall score scale, any organ score ≥ 2 defines the existence of OF. V. Abdominal compartment syndrome (ACS): [13] continuous increase of abdominal pressure (> 20 mmHg, with or without abdominal perfusion pressure ≤ 60 mmHg) and new organ dysfunction/failure (various causes).

#### Local complications

I. Acute peripancreatic fluid collection (APFC): fluid accumulation around the pancreas or organ space without forming a package 4 weeks before AP. II. Acute necrosis collection (ANC): in the early stage of AP, the fluid around the pancreas or organ space is mixed with necrotic tissue around the pancreas or pancreatic parenchyma without forming a complete capsule. III. Walled-off necrosis: occurs 4 weeks after AP, forming a cystic unit that wraps the pancreas or peripancreatic necrotic tissue with a complete envelope. IV. Pancreatic pseudocyst (PPC): develops in the late stage of AP and is characterized by the accumulation of peripancreatic fluid with a complete capsule. V. Infectious pancreatic necrosis (IPN): infection secondary to acute necrotic accumulation or encapsulated necrosis.

### Surgical instruments

In this study, we utilized OLYMPUS TJF 240 V and TJF 260 electronic duodenoscopes, Olympus disposable high-frequency papillotome (KD-V411M-720), The COOK^®^ company’s ribbon guide wire (ACRO-35-450), and The COOK company’s pancreatic duct support (diameter 5–7 Fr, length 4–12 cm).

### Preoperative preparation

I. Upon admission, patients in the stent and non-stent groups received standard conservative treatment, including fasting, rehydration, acid inhibition, enzyme inhibition, nutritional support, and antibiotic therapy tailored to their needs. Analgesic and gastrointestinal decompression methods were employed for patients experiencing severe abdominal pain.

II. Before the operation, patients in both groups underwent a detailed briefing about their medical condition, past medical history, and laboratory and imaging results. A comprehensive evaluation of heart, lung, kidney, and other organ functions was conducted. The operative procedure, potential risks, accidents, and postoperative complications were thoroughly explained to the patients and their families. The potential anesthetic risks during the procedure were also discussed. The patient’s family members must sign the surgical treatment consent form.

### Operation

Stenting of the main pancreatic duct during ERCP was employed to relieve pancreatic duct obstruction, targeting patients experiencing intractable pain due to pancreatic duct stenosis, pancreatic duct stones, or duodenal papillary stenosis.

ERCP treatment process: All patients underwent a preoperative water fast for > 6 h. Intramuscular injection of 10 mg scopolamine butyrate and oral lidocaine gel (10 *g*: 0.2 *g*) was administered 15 min preoperatively to relax the gastrointestinal tract fully. Continuous monitoring of cardiopulmonary function was maintained. After intravenous anesthesia, the patient assumed a left lateral position, and duodenoscopy through the mouth into the gastric cavity proceeded to the descending segment of the duodenum. The position, shape, and opening of the nipple on the inner side of the duodenum were observed. In the pancreatic duct stent group, pancreatic duct intubation was performed using the guide wire guidance method. After successful intubation, fluoroscopy confirmed the guide wire’s alignment with the pancreatic duct. Duodenal papillary sphincter incision knife for nipple micro-incision and suction of the pancreatic duct followed until clear pancreatic juice outflow was observed. The pancreatic duct stent was inserted along the guide wire, followed by the bile duct intubation using the same method. After successful bile duct intubation, duodenal papillary sphincter incision followed along the guide wire, contrast agent injection for cholangiography, stone removal using a stone basket or balloon (ODI’s sphincterotomy if necessary), and mechanical or electrohydraulic lithotripsy shall be performed if needed. Cholangiography was repeated to ensure no residual stones and a nasobiliary drainage tube was placed. In the non-stent group, bile duct intubation was accomplished through the guide wire guidance method. After radiographic confirmation, the catheter was sent along the guide wire, and a contrast agent was injected for observation. Upon confirming the diagnosis of stones, endoscopic removal of stones ensured no residual stones, followed by the placement of a nasobiliary drainage tube.

### Postoperative management

Postoperative management, dietary restrictions, and discharge standards aligned with our previous studies [14, 15]. All patients were treated with diet prohibition, acid inhibition, spasmolysis, fluid replacement, and antibiotic therapy based on changes in inflammatory factors and the patient’s condition. Daily observations of test indices and the patient’s condition were conducted postoperatively. For those with relief of symptoms such as nausea, abdominal distension, and abdominal pain (NRS ≤ 2), and serum amylase decreased to less than three times the upper limit of normal, a return to a soft diet was initiated. The eating time was recorded when the patient returned to a soft diet without stopping oral intake due to symptom recurrence. For patients with moderate or severe AP, enhanced CT scans were performed weekly during hospitalization to evaluate local complications. In the presence of late local complications leading to clinical symptoms, rapid expansion of complications, and secondary infection, and when conservative internal medicine treatment proved ineffective, timely surgical intervention measures were implemented to address the local complications. The choice of intervention method depended on the patient's condition and the location of the local complications. The patient was discharged when oral feeding could not be tolerated without complications and clinical symptoms.

### Follow-up

Patients in both groups underwent biochemical and nasobiliary cholangiography examinations 1–2 weeks after discharge. Once there was no jaundice, abnormal liver function, and residual stones, the nasobiliary drainage tube could be removed. Patients in the stent group underwent enhanced CT 2–3 months after discharge to observe pancreatic recovery and the position of the pancreatic duct stent. If the recovery was satisfactory with no necrosis or effusion, the pancreatic duct stent was removed in the outpatient department.

### Observation indices

I. Time of admission. II. Laboratory indices: Amy, LIP, WBC count, hematocrit (HCT), TBIL, ALT, and creatinine (Cr) at admission and 72 h after admission. III. Efficacy evaluation indicators: recovery time for oral feeding, length of hospital stay, hospitalization cost, late local complications, the incidence of new systemic complications and new OF, and mortality.

### Statistical analysis

The statistical analyses were conducted using the Statistical Package for the Social Sciences version 20.0 (SPSS Inc., Chicago, IL, USA). Non-normally distributed metric variables were analyzed using the Kruskal–Wallis and Mann–Whitney *U*-tests. Unless otherwise stated, values were presented as mean ± standard deviation. For asymmetrically distributed measurement data, it was expressed as the median (interquartile interval) [M (IQR)]. Discrepancies in Amy, HCT, and LIP values between the two groups at admission were noted. Therefore, the analysis of covariance was employed to compare Amy, HCT, and LIP between the two groups at 72 h after admission. Statistical significance was set at *P* < 0.05.

## Results

**Fig. 1:**
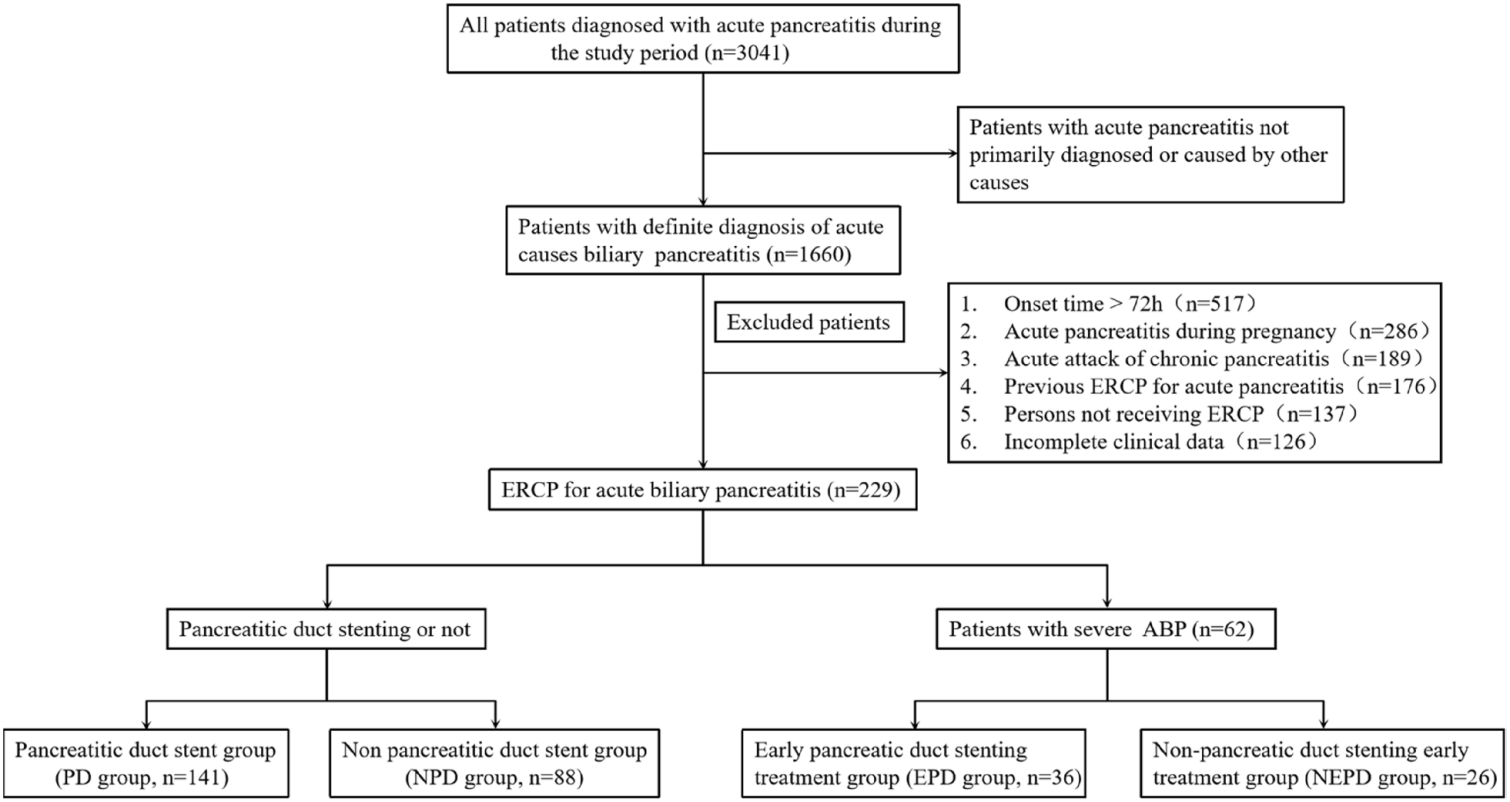
Flowchart illustrating patient inclusion and exclusion

**Fig. 2:**
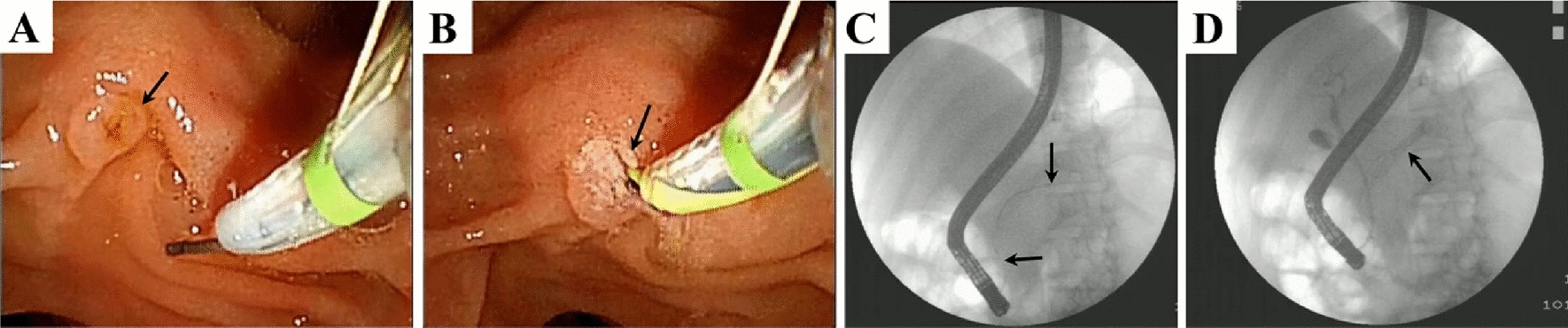
Panoramic depiction of pancreatic duct obstruction and intubation. **A** Duodenal papilla (black arrow). **B** After intubation and aspiration of the pancreatic duct, a substantial amount of obstructive white substances (black arrows) were observed. **C** Fluoroscopy revealed the guide wire (black arrow) following the course of the pancreatic duct. **D** Fluoroscopy demonstrated the successful placement of the pancreatic duct stent (black arrow)

A total of 229 patients with ABP were included, with an average age of (61.86 ± 15.28) years, including 131 men and 98 women. Based on different treatment measures, 141 cases were categorized to the stent group and 88 to the non-stent group. The case screening process is illustrated in Fig. 1. In the stent group, some patients experienced pancreatic duct-blocking substances. During the pancreatic duct stenting procedure, observations revealed the presence of pancreatic ducts, with the scalpel cutting along the nipple, as depicted in Fig. 2.

### Comparison of baseline data between the two groups

No significant differences were observed in gender, age, Acute Physiology and Chronic Health Evaluation (APACHE) II score, ABP severity grade [16], OF, cholangitis or biliary obstruction, and bilirubin levels between the stent and non-stent groups (*P* > 0.05) (Table 1).

**Table 1:** Baseline data of two groups of patients

Variable	Pancreatic duct stent group (*n* = 141)	Non-pancreatic duct stent group (*n* = 88)	P
Age (years, $\overline{\mathrm{x} }$ ± s)	61.53 ± 15.72	63.25 ± 13.76	0.360
Gender (*n*, %)			0.891
Male	80 (56.74%)	51 (57.95%)	
Female	61 (43.26%)	37 (42.05%)	
APACHE II [score, M(IQR)]	8.00 (4.00–12.00)	7.00 (4.00–10.00)	0.400
Moderate and severe ABP (*n*, %)	95 (67.38%)	48 (54.55%)	0.068
OF (*n*, %)	55 (39.01%)	26 (29.55%)	0.158
Cholangitis or biliary obstruction (*n*, %)	86 (60.99%)	44 (50.00%)	0.067
Bilirubin levels	34.91 ± 4.86	35.71 ± 5.23	0.352

### Comparison of treatment effects between the two groups

The results indicated no significant difference in the incidence of complications related to AP between the two groups (*P* > 0.05). The median fasting time and hospital stay in the stent group were significantly shorter than those in the non-stent group (*P* < 0.05). No significant differences were observed in hospitalization expenses and in-hospital mortality between the two groups (*P* > 0.05) (Table 2*)*.

**Table 2:** Comparison of treatment effects and outcomes between the two groups

Variable	Pancreatic duct stent group (*n* = 141)	Non-pancreatic duct stent group (*n* = 88)	P
New complications (*n*, %)	18 (12.77%)	9 (10.22%)	0.675
Whole body (*N*, %)	7 (4.96%)	5 (5.68%)	
Local (*N*, %)	8 (5.76%)	3 (3.41%)	
Newly OF (*n*, %)	3 (2.13%)	1 (1.13%)	
Fasting time [d, M (IQR)]	3 (2–4)%	5 (4–7)	0.001
Length of stay [d, M (IQR)]	5 (3–7)	7 (5–9)	0.001
Hospitalization expenses [10 thousand, M (IQR)]	3.53 (3.06–4.25)	3.52 (2.88–4.19)	0.390
Death (N, %)	1 (0.71%)	1 (1.13%)	0.622

### Comparison of laboratory indices between the two groups after admission and 72 h after admission

When comparing the differences in Amy, WBC, ALT, TBIL, HCT, LIP, Cr, and other laboratory indices between the two groups at admission and 72 h after admission, the results revealed no significant differences in WBC, TBIL, ALT, Cr at admission and 72 h after admission (*P* > 0.05). The levels of AMY at admission and 72 h in the stent group were significantly elevated compared to those in the non-stent group (*P* < 0.05). No significant difference was observed between the two groups (*P* > 0.05). Furthermore, the levels of LIP and HCT in the stent group were significantly higher than those in the non-stent group (*P* < 0.05) (Table 3)*.*

**Table 3:** Comparison of laboratory indexes between the two groups at admission and 72 h after admission

Variable	Pancreatic duct stent group (*n* = 141)	Non-pancreatic duct stent group (*n* = 88)	P
AMY[U/L,M (IQR)]			
On admission	1123.70 (717.00–17912.00)	865.75 (504.75–1316.85)	0.011
72 h	145.70 (97.60–279.05)	107.30 (66.80–168.10)	0.001
Difference	869.00 (506.45–1483.40)	680.70 (388.70–1163.00)	0.085
WBC[× 10^9^/L,M (IQR)]			
On admission	11.40 (8.66–15.35)	10.97 (7.99–15.13)	0.465
72 h	9.23 (6.09–11.93)	7.95 (5.59–10.89)	0.083
Difference	1.88 (-0.88–5.86)	2.15 (-0.07–5.96)	0.462
ALT[U/L,M (IQR)]			
On admission	243.80 (118.50–423.90)	257.10 (150.65–426.50)	0.416
72 h	97.00 (56.55–172.20)	110.00 (70.90–179.15)	0.220
Difference	114.40 (52.55–248.25)	125.90 (51.55–252.40)	0.712
TBIL[umol/L,M (IQR)]			
On admission	68.70 (41.55–114.30)	86.10 (50.15–117.85)	0.117
72 h	30.30 (18.00–46.20)	29.80 (19.50–55.60)	0.439
Difference	37.60 (14.85–69.15)	41.50 (21.15–73.00)	0.216
HCT[%,M (IQR)]			
On admission	43.60 (39.35–47.45)	41.25 (37.83–44.10)	0.001
72 h	38.90 (34.85–42.85)	38.40 (35.70–40.85)	0.422
Difference	4.40 (1.75–7.40)	2.40 (0.80–5.20)	0.001
LIP[IU/L,M (IQR)]			
On admission	8582.50 (4292.50–14294.50)	5222.50 (709.50–9957.00)	0.012
72 h	717.00 (413.00–1525.50)	609.00 (296.25–1174.00)	0.085
Difference	7322.00 (3561.00–13507.00)	3011.00 (70.00–9036.00)	0.006
CR[umol/L,M (IQR)]			
On admission	65.80 (55.95–80.40)	68.00 (52.85–80.47)	0.862
72 h	61.30 (50.60–73.35)	59.50 (50.77–74.52)	0.598
Difference	3.50 (− 1.85–12.70)	5.15 (− 0.42–13.93)	0.206

### Analysis of covariance of some laboratory indices in the two groups

Variations were observed in the values of Amy, HCT, and LIP between the two groups at admission. Due to uneven baseline conditions, an analysis of covariance was employed to assess Amy, HCT, and LIP levels in both groups 72 h after admission. The results demonstrated no significant differences in mean Amy and LIP between the two groups (*P* > 0.05). However, the mean 72-h HCT in the stent group was 38.39% [95% confidence interval (CI) 37.82%–38.96%], which was significantly lower than that in the non-stent group, [39.44% (95% CI 38.70%–40.17%), *P* < 0.05] (Table 4*)*.

**Table 4:** Analysis of covariance of some laboratory indexes of two groups of patients

Variable	Pancreatic duct stent group (*n* = 141)			Non-pancreatic duct stent group (*n* = 88)			*F*	*P*
	M	SD	95%CI	M	SD	95%CI		
AMY	223.87	16.84	190.67–257.07	184.68	21.70	141.92–227.45	2.03	0.15
LIP	1288.17	178.20	935.69–1640.64	1617.45	294.41	1035.12–2199.78	0.90	0.34
HCT	38.39	0.28	37.82–38.96	39.44	0.37	38.70–40.17	4.83	0.02

## Discussion

Pancreatic duct stents, commonly used for pancreatic drainage, have gained recognition in treating chronic pancreatitis, pancreatic duct division, and complications of AP (such as pancreatic duct rupture and pancreatic pseudocyst). Moreover, pancreatic duct stents are crucial in preventing pancreatitis following ERCP. During ERCP operation, factors such as duodenal papilla intubation can induce spasm and edema of the papillary sphincter, impeding the smooth discharge of pancreatic juice. The utilization of pancreatic duct stents facilitates the adequate drainage of pancreatic fluid, thereby preventing complications in certain patients post-ERCP [17]. There is a lack of consensus regarding the treatment approach and optimal timing for stent placement in ABP. This study aimed to address this gap by reviewing and analyzing clinical data from our pancreatitis center, focusing on patients with ABP treated using pancreatic duct stents and comparing them with those treated without stents to explore the efficacy and feasibility of this intervention.

The study included 229 patients with ABP who underwent ERCP. The median ALT at admission was 245.90 U/L, indicating liver injury. Liver function impairment is a common complication of ABP, attributed to the overactivation of pancreatin, cytokine release, and disturbances in liver microcirculation. ABP can induce hepatocyte degeneration and necrosis as overactivated pancreatin and bacterial toxins reach the liver through the portal vein system. In addition, most ABPs have bile duct obstruction and poor bile excretion. Bacterial toxins, released during secondary infections, can cause bacteremia, directly impacting the liver via the bloodstream [18]. Therefore, endoscopic treatment alleviated biliary obstruction and reduced bile duct pressure, followed by nasobiliary drainage tube placement, facilitated smooth bile drainage, terminated the inflammatory reaction, and prevented further liver damage.

Moreover, some scholars have reported that the duration of ampullary obstruction in patients with ABP is linked to liver function impairment and positively correlates with the severity of AP. Prolonged ampullary obstruction exacerbates the condition, contributing to pancreatic bleeding and necrosis. Effectively addressing the block within 24 h can prevent patient deterioration. Beyond 48 h, the risk of pancreatic tissue necrosis increases, resulting in critical conditions, challenging treatment, poor prognosis, and slow patient recovery [19]. Therefore, under favorable conditions, it is crucial to comprehensively evaluate the patient's condition, identify the surgical indications, and promptly relieve ampullary obstruction in patients with obstruction. This approach significantly contributes to enhanced patient recovery. In this study, leukocyte count, TBIL, and serum Amy and LIP levels showed significant decreases in all patients 72 h after admission, returning to normal levels. These findings underscore the positive impact of ERCP in relieving bile duct obstruction and ensuring bile emptying in the rehabilitation of patients with ABP. Consequently, industry guidelines advocate for early intervention in those patients with ABP with biliary obstruction or acute cholangitis to alleviate obstruction and improve clinical outcomes [10, 20].

While the effectiveness of endoscopic treatment for ABP is well-established, questions linger regarding the comparative benefits of pancreatic stent implantation for patients. Patients were categorized into two groups based on distinct treatment approaches to delve into the efficacy of pancreatic stent implantation during ERCP in ABP treatment. No significant differences between the two groups were observed in WBC and ALT before and after admission (*P* > 0.05). Differences in Amy, HCT, and LIP existed between the groups upon admission, possibly stemming from prior treatment at other hospitals for some patients. Analysis of covariance was employed to compare Amy, HCT, and LIP levels between the two groups 72 h after admission to mitigate the impact of this baseline variation on this efficacy assessment. The results revealed no significant differences in Amy and LIP at 72 h; however, the HCT at 72 h in the stent group was significantly lower than in the non-stent group. The elevation of HCT during ABP may be attributed to abnormal trypsin activation, leading to the release of numerous cell inflammations, thereby increasing capillary wall permeability and causing substantial plasma-like-fluid exudation from the circulatory system into the abdominal cavity or tissue space [21]. Research has indicated that using HCT alone to evaluate the condition upon admission yields sensitivity and negative predictive value equivalent to the Ranson score after 48 h. However, its specificity, positive predictive value, and overall accuracy are relatively low. HCT demonstrates a significant correlation with the Balthazar score, length of stay in the ICU, and total length of hospitalization. It can serve as an indicator for predicting the condition and assessing the severity of patients with AP [22]. Subsequently, patients in the stent group exhibited a more rapid decrease in HCT, possibly attributed to unimpeded drainage of pancreatic juice, reducing the interaction between trypsin and substrate. This reduction weakened trypsin’s impact on vascular permeability, resulting in a relatively diminished plasma exudation. Thereby, the patient’s condition improved after pancreatic duct stent implantation, mitigating disease progression to some extent.

In this investigation, patients in the stent group experienced significantly shorter hospitalization periods than those in the non-stent group. This outcome may be attributed to symptom improvement post-stent implantation, facilitating a swift eating recovery and promoting gastrointestinal tract restoration. Gastrointestinal tract recovery holds paramount importance for patients with AP, given that the majority of them suffer from varying degrees of gastrointestinal dysfunction. This dysfunction is primarily caused by systemic inflammation-induced inadequate blood supply and oxygen to the gastrointestinal mucosa, frequently resulting in symptoms such as abdominal distension and intestinal paralysis [23]. Persistent gastrointestinal dysfunction increases the risk of intestinal flora translocation and secondary infection [24]. Some studies propose that early (within 48 h) initiation of enteral nutrition and intervention measures, such as enhancing intestinal microcirculation, alleviating abdominal pain, reducing intestinal edema, providing intestinal decompression, and cleansing, contribute to restoring intestinal function [25]. Initiating oral feeding or enteral nutrition promptly upon restoring intestinal function is crucial to minimize the risk of infection effectively [26]. However, the advantages of early resumption of oral feeding surpass those of enteral nutrition. Consequently, patients in the stent group experience shorter hospitalization times and reduced costs. Research has indicated that continuous high pressure in the pancreatic duct can diminish pancreatic blood flow, leading to pancreatic ischemia—a significant factor in pancreatic edema, bleeding, and necrosis [27]. Elevated pancreatic duct pressure may also facilitate the infiltration of inflammatory mediators into the pancreatic parenchyma, triggering a cascade reaction and causing damage to the pancreatic tissue. In our study, the smooth insertion of a pancreatic duct stent facilitated the drainage of pancreatic juice, alleviating pancreatic duct hypertension and preventing further harm to the pancreas, thereby contributing to favorable patient outcomes. Compared to non-stent treatments, this approach significantly shortens the patients’ course.

Selecting a pancreatic duct stent involves careful consideration of its inner diameter and length. The 5 Fr, 7 Fr, or 8.5 Fr stents are generally employed for small pancreatic ducts. Patients with evident main pancreatic ductal expansion or chronic pancreatitis may benefit from 10 Fr–11.5 Fr stents or 5–7 Fr double stents. A 3 Fr pancreatic duct stent is commonly used for ERCP to prevent ERCP-related pancreatitis after Oddi’s sphincterotomy, typically self-dislodging within 1–2 weeks post-ERCP [28]. In our study, the choice of pancreatic duct stents aligned with the degree of pancreatic duct expansion, utilizing 5 Fr stents for patients without expansion and 7 Fr stents for those with ductal expansion. Pancreatic duct stent displacement following implantation is a recognized long-term complication. Johanson et al. [29] documented migration rates of 5.2% to the proximal end [14] and 7.5% to the distal end [20] among 267 patients with pancreatic duct stent implantation. In this study, with 141 patients in the pancreatic duct stent group, a portion of the pancreatic duct stents naturally dislodged, passing through the intestine and subsequently expelled with feces. Some patients independently removed the stent during outpatient visits, utilizing a 5F spiral basket retractor to cover the stent’s side and extract it without requiring additional surgical intervention. Various scholars have raised concerns regarding the potential risk of bacterial entry into the pancreatic duct with the placement of small-diameter pancreatic duct stents during recurrent attacks of ABP. This risk may be attributed to transforming the aseptic necrotic pancreatic microenvironment into infectious necrosis or abscess formation. Intraoperative sterility principles were strictly adhered to mitigate this risk by refraining from injecting contrast agents into the pancreatic duct or using minimal amounts during intubation. Extensive incisions of the nipple sphincter were avoided to preserve the normal barrier function of the major duodenal papilla and minimize the likelihood of bacterial migration. No considerable increase in sepsis complications associated with pancreatic stent placement was observed in the stent group.

This study reported no severe postoperative complications such as gastrointestinal bleeding, perforation, or severe pancreatitis following ERCP in both groups. Our approach of avoiding extensive incisions of the Oddi sphincter intraoperatively and opting for smaller incisions to facilitate endoscopic procedures for most patients, contributed to preventing certain endoscopic complications linked to large Oddi sphincter incisions. In placing pancreatic duct stents, we employed the guide wire guidance method and double guide wire intubation method to minimize repeated intubation, thereby defining the direction of bile duct opening. This approach facilitates endoscopic stone removal and nasal bile duct placement. Moreover, previous studies have highlighted that more experienced endoscopists exhibit lower complication rates and higher success rates [30], a phenomenon observed in this study where seasoned professionals conducted all ERCP procedures with an annual average of over 500 ERCP operations per endoscopist and a cumulative total of > 1000 pancreatic duct intubations. This proficiency contributed to the avoidance of most complications. It is crucial to note that patients with severe AP may present with severe duodenal and ampullary edema in the early stages. Lack of ERCP and pancreatic duct intubation experience in such cases can lead to serious adverse events [31]. Therefore, we recommend that experienced ERCP centers and operators carry out ERCP operations for SAP.

In summary, pancreatic stent implantation, compared to non-pancreatic stent surgery, significantly shortens the time for oral feeding and hospitalization in patients with ABP. This approach fosters the swift recovery of patients with ABP, suggesting the safety and efficacy of pancreatic stent implantation in ABP treatment. Moreover, this study sheds light on the potential role of pancreatic duct obstruction/hypertension in ABP pathogenesis.

Limitations of the study: This retrospective analysis is a single-center study, lacking the rigorous experimental design in multi-center randomized controlled trials. Consequently, the results have a degree of bias, rendering the level of evidence low. Given the limited cases in this study, further validation through multi-center, large-sample studies is essential. Despite these limitations, this research offers novel insights into the treatment and analysis of ABP.

## Data Availability

The datasets generated during and/or analyzed during the current study are available from the corresponding author on reasonable request.
